# Commentary on “Epigenome-wide analysis across the development span of pediatric acute lymphoblastic leukemia: backtracking to birth”

**DOI:** 10.1186/s12943-024-02220-7

**Published:** 2025-01-11

**Authors:** Emma Raitoharju, Saara Marttila

**Affiliations:** 1 Molecular Epidemiology (MOLE), Faculty of Medicine and Health Technology, Tampere University, Tampere, Finland; 2 Fimlab Laboratories, Tampere, Finland; 3 Tampere University Hospital, Wellbeing Services County of Pirkanmaa, Tampere, Finland

**Keywords:** nc886, VTRNA2-1, Paediatric leukaemia, Epigenetics, DNA methylation, Imprinting

## Abstract

VTRNA2-1 is a polymorphically imprinted locus. The proportion of individuals with a maternally imprinted VTRNA2-1 locus is consistently approximately 75% in populations of European origin, with the remaining circa 25% having a non-methylated VTRNA2-1 locus. Recently, VTRNA2-1 hypermethylation at birth was suggested to be a precursor of paediatric acute lymphoblastic leukaemia with biomarker potential. The results presented by Ghantous et al. [1] allow for an alternative interpretation to what the authors discussed, and we argue that the observed methylation difference at birth is due to an uneven distribution of imprinted and non-methylated individuals among the cases and controls, with all individuals presenting normative physiological VTRNA2-1 methylation levels. In addition, the notable interindividual variation arising from the polymorphic imprinting in VTRNA2-1 methylation levels calls into question the validity of VTRNA2-1 methylation as a biomarker.

**Supplementary Information:**

The online version contains supplementary material available at 10.1186/s12943-024-02220-7.

## Main body

**Fig. 1:**
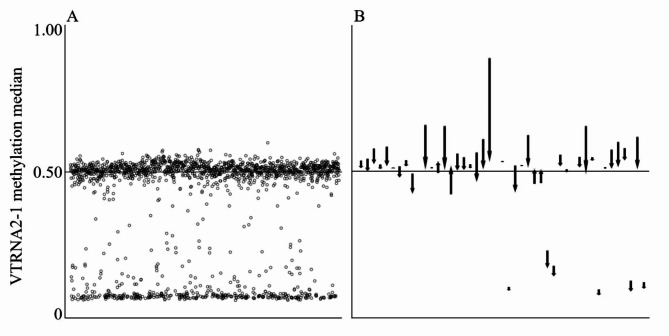
In **A**, a representative example of the median methylation level of the VTRNA2-1 locus in a healthy population cohort (GSE105018 [13] from GEO [14]). CpGs included in the VTRNA2-1 median are provided in Supplementary Methods. Each dot represents one individual, *n* = 1,658. Notable interindividual variation can be observed, and individuals can be classified into those with an imprinted VTRNA2-1 locus (methylation beta-value > 0.40, *n* = 1,226, 73.9%) and to those with a non-methylated VTRNA2-1 locus (methylation beta-value < 0.20, *n* = 395, 23.8%). A small minority (*n* = 37, 2.2%) presents intermediate values, as expected [6]. Scatterplots for each of the 14 CpG sites located at VTRNA2-1 are provided in Supplementary Fig. 1. Panel **B** demonstrates the change in the median methylation level of VTRNA2-1 between samples at diagnosis of B-ALL (start of the arrow) and at remission (end of the arrow) (GSE38235 [15] from GEO [14], this dataset is also included in Ghantous et al., [1]). Each arrow represents one individual, *n* = 43. In most individuals, the at-diagnosis sample is hypermethylated in comparison to the sample taken while in remission, but for some individuals, hypomethylation of comparable magnitude can be observed. Notably, the magnitude of the change associated with cancer is, for most individuals, modest in comparison to the interindividual variation observed between the individuals with an imprinted and those with a non-methylated VTRNA2-1 locus. Individual CpGs at VTRNA2-1 are presented in Supplementary Fig. 2

VTRNA2-1 (also known as nc886) is a polymorphically imprinted locus in 5q31.1, encoding a 102-nucleotide-long non-coding RNA that is cleaved into two short RNAs with low efficiency [2, 3]. In approximately 75% of individuals of European origin, the maternal allele of VTRNA2-1 is methylated, and these individuals present with 50% methylation at the locus. In the remaining approximately 25% of the population, both the maternal and the paternal allele are non-methylated, and these individuals present with a methylation level close to 0% (Fig. 1A [4]). The methylation status of VTRNA2-1 has been shown to remain stable through life [5, 6] and to be similar in the majority of the somatic tissues of an individual [4, 6, 7]. The expression levels of VTRNA2-1 RNAs are strongly associated with the methylation pattern of the locus [6, 8]; in individuals with a non-methylated VTRNA2-1 locus, the levels of VTRNA2-1 RNAs are approximately two-fold compared to individuals with an imprinted VTRNA 2 − 1 locus [6, 9]. VTRNA2-1 methylation changes, as well as the expression of VTRNA2-1 RNAs, have been shown to be associated with various types of cancer, and both hypo- and hypermethylation of VTRNA2-1 have also been associated with the cancer prognosis [2, 10]. Notably, even with the cancer-associated changes in VTRNA2-1 methylation, the bimodal methylation pattern is often retained [2].

Recently, Ghantous et al. [1] identified CpG sites located at the VTRNA2-1 locus to be differentially methylated at birth between individuals who later developed paediatric pre-B acute lymphoblastic leukaemia (B-ALL; cases) and those who remained healthy (controls). This was interpreted as hypermethylation of the locus in the cases, as an epigenetic precursor for B-ALL, and the authors further suggested that VTRNA2-1 methylation could be utilized as a biomarker or a therapy target for B-ALL [1]. However, taking into account the polymorphic imprinting pattern of VTRNA2-1 allows for an alternative interpretation of the results, namely that the observed difference in methylation levels at birth is due to an uneven distribution of individuals with an imprinted or non-methylated VTRNA2-1 locus between cases and controls, with all individuals presenting a physiological methylation pattern at the locus.

The VTRNA2-1 methylation levels at birth in MoBa, one of the two discovery cohorts utilized by Ghantous et al. [1], present the expected bimodal pattern, with approximately ¼ of the cohort presenting very low methylation levels and some ¾ presenting a methylation level of approximately 50% (Supplementary Fig. 6C in Ghantous et al. [1]). No distinct methylation pattern for cases can be observed, as in the heatmap the cases do not cluster together, but are distributed among the controls by hierarchical clustering (Supplementary Fig. 6C in Ghantous et al. [1]) and the range of methylation values in CpGs located at VTRNA2-1 is comparable between cases and controls at birth (Fig. 1F in Ghantous et al. [1]). In order to report the hypermethylation of the VTRNA2-1 locus convincingly, the methylation level of the tissue or condition of interest would need to be compared to the intrinsic imprinting status of the individual, or it would have to be demonstrated that the methylation level is different from the expected physiological methylation level, either imprinted or non-methylated.

Only two of the 22 cases (9%) in MoBa show methylation levels indicative of a non-methylated VTRNA2-1 locus (Supplementary Fig. 6C in Ghantous et al. [1]), which is a considerably lower proportion than what is reported for the MoBa cohort as a whole or the 25% reported for population cohorts [4]. Unfortunately, data from two other at-birth cohorts utilized by Ghantous et al. [1] are not freely available, nor are the methylation levels presented in the article, to enable an analysis of the proportion of individual with a non-methylated VTRNA2-1 locus among the cases. However, the underrepresentation of individuals with a non-methylated VTRNA2-1 locus among cases in MoBa suggests that individuals with a non-methylated VTRNA2-1 locus could be protected against B-ALL. Whether the intrinsic imprinting status of VTRNA2-1 is associated with the risk of B-ALL warrants further study. Previously, this has been shown for prostate, breast and pancreatic cancer [2, 11].

As additional evidence for the utility of VTRNA2-1 methylation as a B-ALL biomarker, Ghantous et al. [1] report that CpGs located at VTRNA2-1 are hypermethylated in B-ALL tissues at diagnosis (as compared to controls or to remission) and that hypermethylation at two CpGs at VTRNA2-1 was associated with poorer survival in B-ALL. However, at the diagnosis of B-ALL, the bimodal methylation pattern is retained, and approximately ¼ of individuals present methylation levels close to 0%, while ¾ present methylation levels close to 50% (Supplementary Fig. 6D in Ghantous et al. [1]). When comparing samples at diagnosis and in remission, it can be observed that the change in VTRNA2-1 methylation level associated with malignancy is modest in magnitude when compared to the interindividual differences observed due to the polymorphic imprinting status (Fig. 1B). An ideal biomarker would have both high specificity and high sensitivity, the ability to separate cases from controls accurately [12]. As VTRNA2-1 methylation levels are comparable in cases and controls at birth, and the change associated with malignancy is modest in comparison to the interindividual variation, VTRNA2-1 methylation level cannot be considered a promising biomarker for B-ALL.

## Conclusion

When the polymorphic imprinting pattern and the considerable physiological interindividual variation in VTRNA2-1 methylation is taken into account, the data presented by Ghantous et al. [1] do not, in our opinion, support the interpretation that VTRNA2-1 hypermethylation is a precursor of paediatric B-ALL, nor do they support the role of VTRNA2-1 methylation as a biomarker for B-ALL. There are many lines of evidence suggesting that VTRNA2-1 methylation and VTRNA2-1 RNA expression are associated with the incidence and progression of cancer [2, 3, 9–11], and this may also be true for B-ALL. However, not accounting for the polymorphic imprinting pattern of VTRNA2-1 will lead to erroneous conclusions and potentially prevents us from uncovering the true role of VTRNA2-1 in cancer.

## Electronic supplementary material

Below is the link to the electronic supplementary material.

Supplementary Material 1

## Data Availability

The datasets utilized are available from the Gene Expression Omnibus (GEO [14]) under accession numbers GSE105018 [13] and GSE38235 [15].
