# Supplementary material for: Commentary on “Epigenome-wide analysis across the development span of pediatric acute lymphoblastic leukemia: backtracking to birth”
Source: Mol Cancer. 2025 Jan 11;24:8. doi: 10.1186/s12943-024-02220-7 (PMC11724579; doi:10.1186/s12943-024-02220-7)
Supplement: Supplementary file 1 — Supplementary Material 1 [file 12943_2024_2220_MOESM1_ESM.docx]

**Supplementary File**

Commentary on “Epigenome-wide analysis across the development span of pediatric acute lymphoblastic leukemia: backtracking to birth”

Emma Raitoharju^1,2^ & Saara Marttila^1,3^

^1^Molecular Epidemiology (MOLE), Faculty of Medicine and Health Technology, Tampere University, Tampere, Finland

^2^Fimlab Laboratories**,** Tampere, Finland

^3^Tampere University Hospital, Wellbeing Services County of Pirkanmaa, Tampere, Finland

Correspondence: saara.marttila@tuni.fi, emma.raitoharju@tuni.fi

Supplementary Methods, page 2

Supplementary Figures, page 3

References, page 6

**Supplementary Methods**

*Data sets*

Datasets GSE105018 (Hannon et al., 2018; Marzi et al., 2018) and GSE38235 (Busche et al., 2013) were retrieved from Gene Expression Omnibus (GEO, Edgar et al., 2002; Barrett et al., 2013). For both datasets, pre-processed data available in GEO was downloaded and used as such.

*VTRNA2-1 methylation level*

From the GEO datasets, 14 CpGs (cg07158503, cg04515200, cg13581155, cg11978884, cg11608150, cg06478886, cg04481923, cg18678645, cg06536614, cg25340688, cg26896946, cg00124993, cg08745965, cg18797653) located in the VTRNA2-1 locus were extracted (Marttila et al., 2021; Marttila et al., 2022). Median methylation level of these CpGs were calculated to represent the whole VTRNA2-1 locus. Median methylation level for whole VTRNA2-1 locus is presented in the main body of text, each of the 14 CpGs are presented individually in the Supplementary Figures 1 and 2.

**Supplementary Figures**


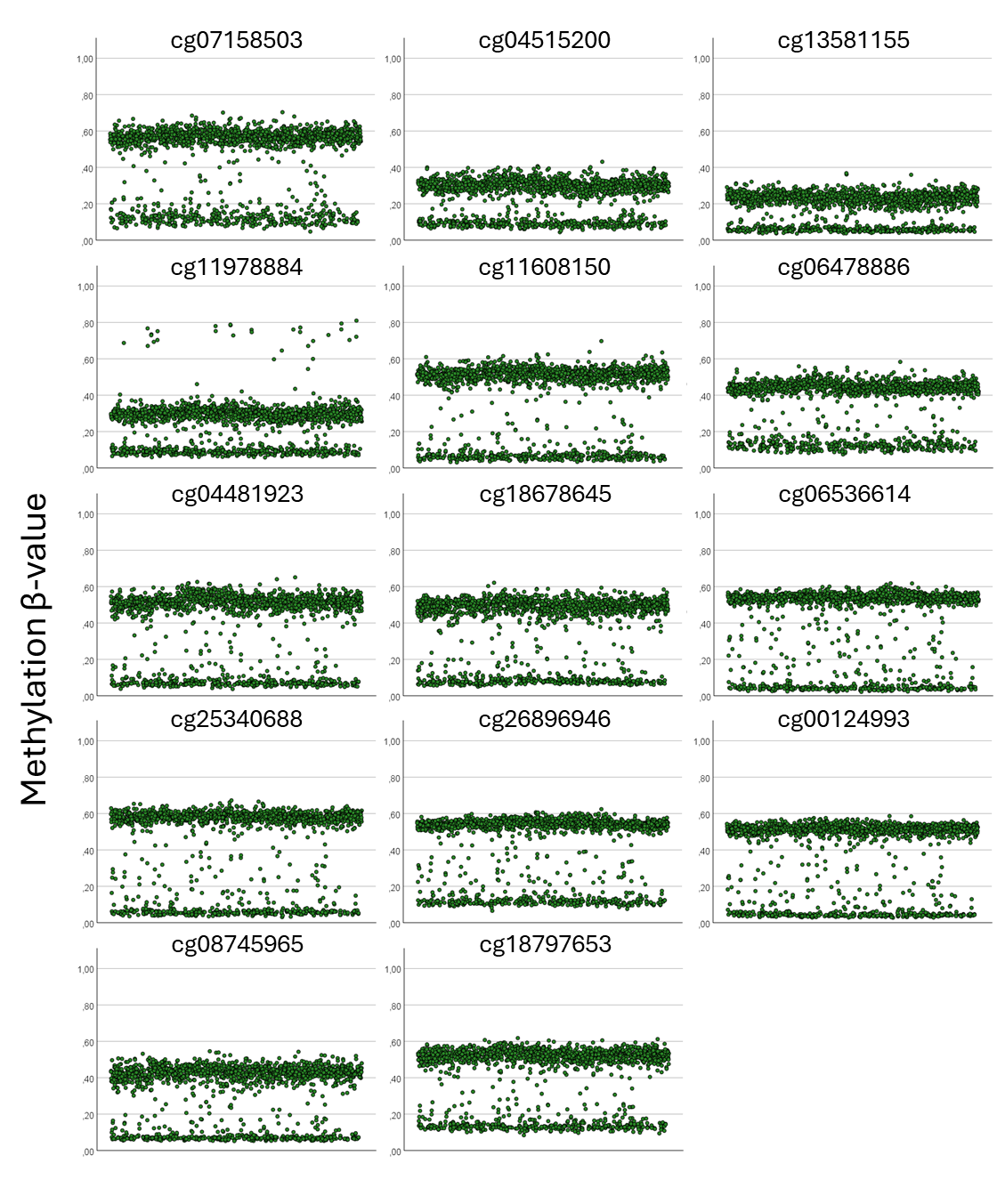


**Supplementary Figure 1.** Methylation level at individual CpG sites located in the polymorphically imprinted VTRNA2-1 (nc886). Each dot represents one individual, data from GSE105018, n=1658. For each CpG, a clear dichotomous pattern of DNA methylation can be observed, corresponding to imprinted and non-methylated status of VTRNA2-1.


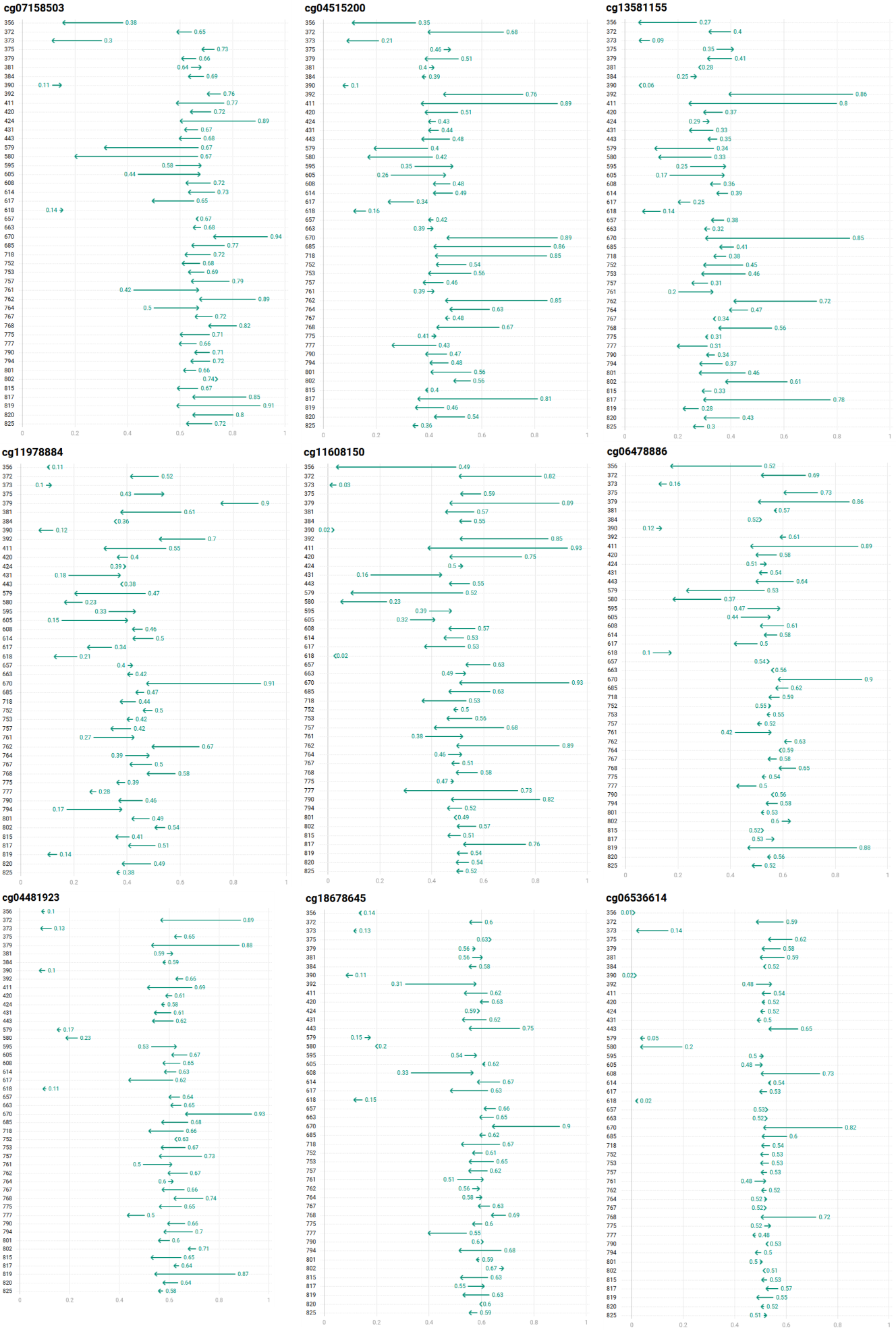


**Supplementary Figure 2.** Continued on next page.


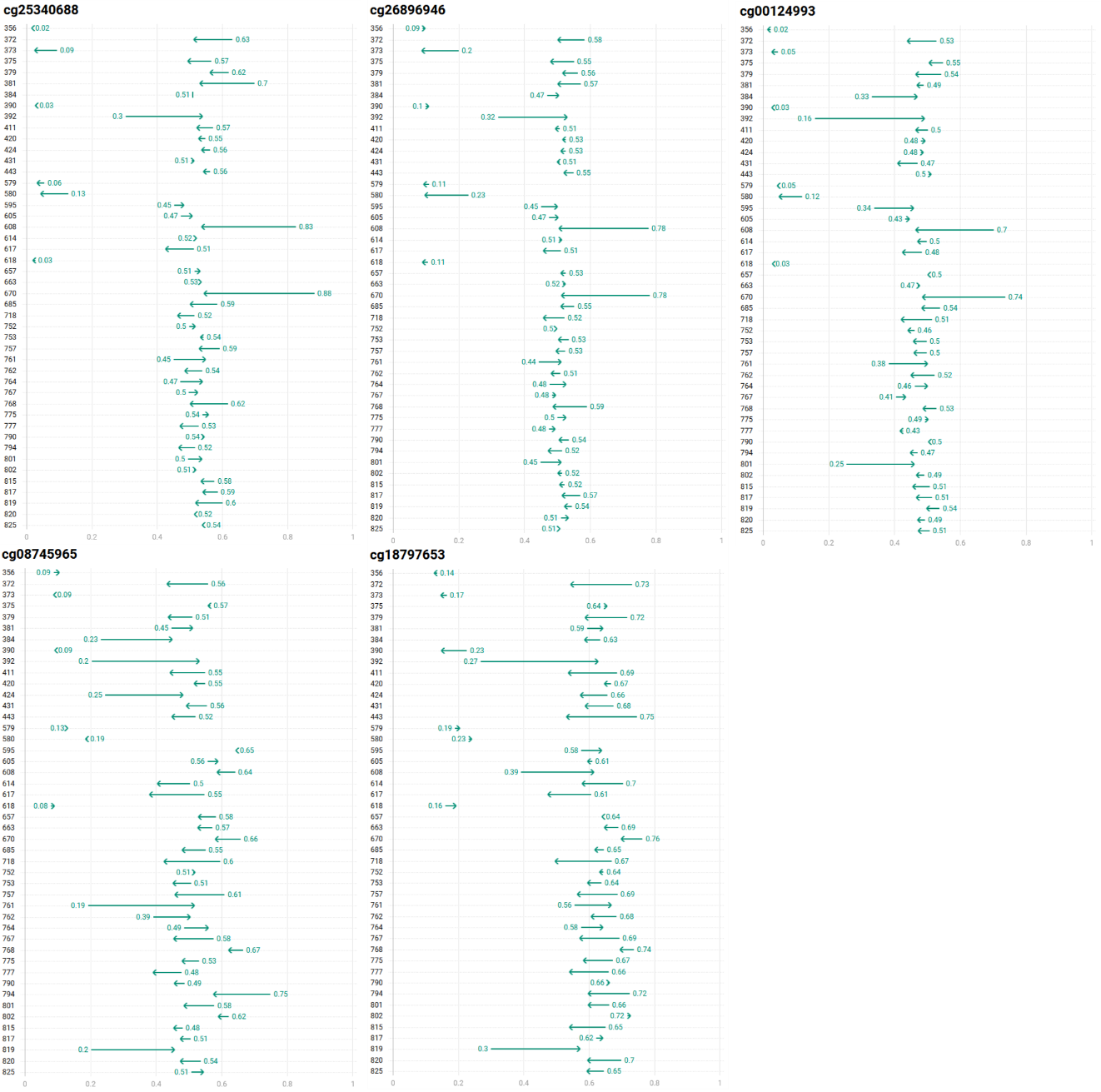


**Supplementary Figure 2.** Change in methylation level in 14 CpGs located in VTRNA2-1 locus between tumor samples and samples at remission in pre-B ALL. Start of the arrow is the tumor sample, end of the arrow is the sample at remission. Data from GSE38235 (Busche et al., 2013). For majority of CpGs in majority of individuals, the cancer associated change is more modest as compared to the interindividual variation. In Ghantous et al., (2024) methylation level at diagnosis in cg00124993 and cg04481923 was reported to be associated with B-ALL survival. Figures created with Datawrapper, www.datawrapper.de.

**References**

Barrett T, Wilhite SE, Ledoux P, Evangelista C, Kim IF, Tomashevsky M, Marshall KA, Phillippy KH, Sherman PM, Holko M, Yefanov A, Lee H, Zhang N, Robertson CL, Serova N, Davis S, Soboleva A. NCBI GEO: archive for functional genomics data sets--update. Nucleic Acids Res. 2013 Jan;41(Database issue): D991-5. doi:10.1093/nar/gks1193.

Busche S, Ge B, Vidal R, Spinella JF, Saillour V, Richer C, Healy J, Chen SH, Droit A, Sinnett D, Pastinen T. Integration of high-resolution methylome and transcriptome analyses to dissect epigenomic changes in childhood acute lymphoblastic leukemia. Cancer Res. 2013 Jul 15;73(14):4323-36. doi: 10.1158/0008-5472.CAN-12-4367.

Edgar R, Domrachev M, Lash AE. Gene Expression Omnibus: NCBI gene expression and hybridization array data repository. Nucleic Acids Res. 2002 Jan 1;30(1):207-10. doi:10.1093/nar/30.1.207.

Ghantous A, Nusslé SG, Nassar FJ, Spitz N, Novoloaca A, Krali O, Nickels E, Cahais V, Cuenin C, Roy R, Li S, Caron M, Lam D, Fransquet PD, Casement J, Strathdee G, Pearce MS, Hansen HM, Lee HH, Lee YS, de Smith AJ, Sinnett D, Håberg SE, McKay JA, Nordlund J, Magnus P, Dwyer T, Saffery R, Wiemels JL, Munthe-Kaas MC, Herceg Z. Epigenome-wide analysis across the development span of pediatric acute lymphoblastic leukemia: backtracking to birth. Mol Cancer. 2024 Oct 23;23(1):238. doi: 10.1186/s12943-024-02118-4.

Hannon E, Knox O, Sugden K, Burrage J, Wong CCY, Belsky DW, Corcoran DL, Arseneault L, Moffitt TE, Caspi A, Mill J. Characterizing genetic and environmental influences on variable DNA methylation using monozygotic and dizygotic twins. PLoS Genet. 2018 Aug 9;14(8):e1007544. doi: 10.1371/journal.pgen.1007544.

Marttila S, Tamminen H, Rajić S, Mishra PP, Lehtimäki T, Raitakari O, Kähönen M, Kananen L, Jylhävä J, Hägg S, Delerue T, Peters A, Waldenberger M, Kleber ME, März W, Luoto R, Raitanen J, Sillanpää E, Laakkonen EK, Heikkinen A, Ollikainen M, Raitoharju E. Methylation status of *VTRNA2-1*/*nc886* is stable across populations, monozygotic twin pairs and in majority of tissues. Epigenomics. 2022 Sep;14(18):1105-1124. doi: 10.2217/epi-2022-0228.

Marttila S, Viiri LE, Mishra PP, Kühnel B, Matias-Garcia PR, Lyytikäinen LP, Ceder T, Mononen N, Rathmann W, Winkelmann J, Peters A, Kähönen M, Hutri-Kähönen N, Juonala M, Aalto-Setälä K, Raitakari O, Lehtimäki T, Waldenberger M, Raitoharju E. Methylation status of nc886 epiallele reflects periconceptional conditions and is associated with glucose metabolism through nc886 RNAs. Clin Epigenetics. 2021 Jul 22;13(1):143. doi: 10.1186/s13148-021-01132-3.

Marzi SJ, Sugden K, Arseneault L, Belsky DW, Burrage J, Corcoran DL, Danese A, Fisher HL, Hannon E, Moffitt TE, Odgers CL, Pariante C, Poulton R, Williams BS, Wong CCY, Mill J, Caspi A. Analysis of DNA Methylation in Young People: Limited Evidence for an Association Between Victimization Stress and Epigenetic Variation in Blood. Am J Psychiatry. 2018 Jun 1;175(6):517-529. doi: 10.1176/appi.ajp.2017.17060693. Epub 2018 Jan 12.
